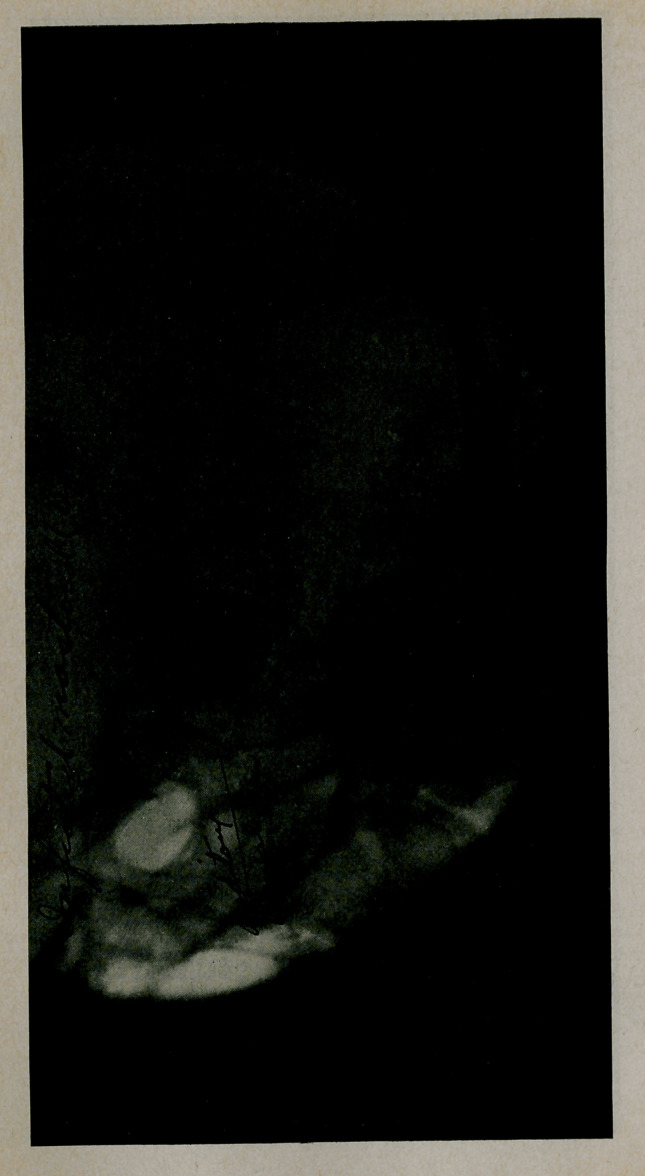# The Value of Radiographs in the Diagnosis of Mastoditis

**Published:** 1913-12

**Authors:** 


The Value of Radiographs in the Diagnosis of Mastoiditis. J. M. Ingersoll, Cleveland, *Cleveland Medical Journal*, September, 1913, reports three cases, with radiographs of one, as follows:

"Case III.—Male, thirty years old. Acute otitis media for ten days. Discharge from ear gradually decreasing. Some granulation tissue projection through the perforation in the drum membrane. Some swelling of the posterior superior part of the canal wall. No pain over the mastoid. No spontaneous nystagmus. Turning test normal. Temperature normal.

A radiograph showed that all of the mastoid cells were infected, that the sinus was exposed just below and posterior to the mastoid antrum, also that the sinus was situated well forward close to the antrum and that the mastoid bone was very small.

All of these conditions were confirmed by the operative findings. The recovery was a normal one.

The stereoscopic pictures in these cases were similar to those in many other cases and they illustrate the decided value of such pictures in determining the condition of the mastoid bone and in making a positive diagnosis of mastoiditis or exposure of the brain or the sinus by necrosis, or the exclusion of such conditions."

The cuts are reproduced by courtesy of the editorial staff.

## Figures and Tables

**Figure f1:**
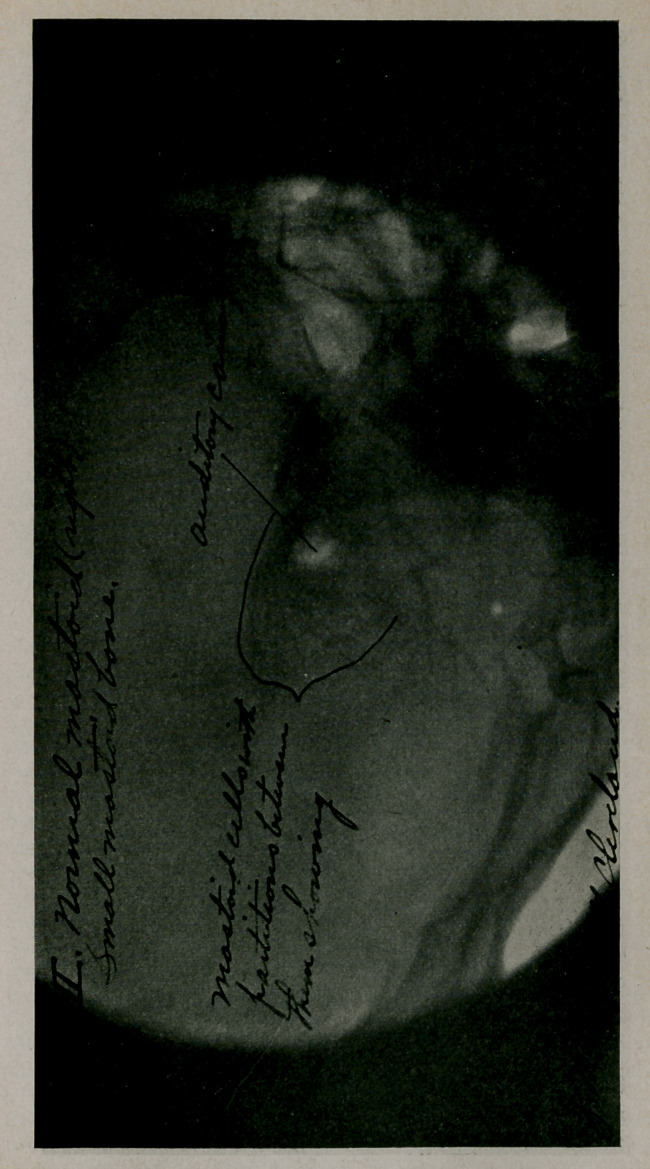


**Figure f2:**